# Psychological effects of remote-only communication among reference persons of ICU patients during COVID-19 pandemic

**DOI:** 10.1186/s40560-020-00520-w

**Published:** 2021-01-09

**Authors:** Jessy Cattelan, Sara Castellano, Hamid Merdji, Jean Audusseau, Baptiste Claude, Léa Feuillassier, Sibylle Cunat, Marc Astrié, Camille Aquin, Guillaume Buis, Edgar Gehant, Amandine Granier, Hassiba Kercha, Camille Le Guillou, Guillaume Martin, Kevin Roulot, Ferhat Meziani, Olivier Putois, Julie Helms

**Affiliations:** 1 Service de Médecine Intensive-Réanimation, Nouvel Hôpital Civil, Hôpitaux universitaires de Strasbourg, Strasbourg, France; 2 SuLiSoM UR 3071, Faculté de Psychologie, Université de Strasbourg, Strasbourg, France; 3 Unité Médico-Judiciaire, Institut Médico-Légal, Hôpital Raymond-Poincaré, Garches, France; 4 INSERM, UMR 1260, Regenerative Nanomedicine, Fédération de Médecine Translationnelle de Strasbourg, Université de Strasbourg (UNISTRA), Strasbourg, France; 5 LPC UR 4440, Faculté de Psychologie, Université de Strasbourg, Strasbourg, France; 6 Département de Psychiatrie, Santé Mentale et Addictologie, Hôpitaux Universitaires de Strasbourg, Strasbourg, France; 7 Institut Thématique Interdisciplinaire TRANSPLANTEX NG, Université de Strasbourg, Institut d’Immunologie et d’Hématologie, Strasbourg, France; 8 ImmunoRhumatologie Moléculaire, INSERM UMR_S1109, LabEx TRANSPLANTEX, Centre de Recherche d’Immunologie et d’Hématologie, Faculté de Médecine, Fédération Hospitalo-Universitaire OMICARE, Fédération de Médecine Translationnelle de Strasbourg, Université de Strasbourg (UNISTRA), Strasbourg, France

**Keywords:** COVID-19; Reference person; Relative; Anxiety; Depression; Distress

## Abstract

**Background:**

During COVID-19 pandemic, visits have been prohibited in most French ICUs. Psychological effects, for reference persons (RPs), of remote-only communication have been assessed.

**Methods:**

All RPs of patients referred to ICU for COVID-19 were included. HADS, IES-R, and satisfaction were evaluated at admission, discharge/death, and 3 months. At 3 months, a psychologist provided a qualitative description of RPs’ psychological distress.

**Results:**

Eighty-eight RPs were included. Prevalence of anxiety and depression was 83% and 73% respectively. At 3 months, lower HADS decrease was associated with patient death/continued hospitalization, and/or sleeping disorders in RPs (*p* < 0.01). Ninety-nine percent RPs felt the patient was safe (9 [7; 10]/10 points, Likert-type scale), confident with caregivers (10 [9; 10]/10 points), and satisfied with information provided (10 [9; 10]/10 points). All RPs stressed the specific-type of “responsibility” associated with being an RP in a remote-only context, leading RPs to develop narrow diffusion strategies (67%) and restrict the array of contacted relatives to a very few and/or only contacting them rarely. 10 RPs (30%) related the situation to a prior traumatic experience.

**Conclusion:**

RPs experienced psychological distress and reported that being an RP in a remote-only communication context was a specific responsibility and qualified it as an overall negative experience.

**Trial registration:**

NCT04385121. Registered 12 May 2020. https://clinicaltrials.gov/.

**Supplementary Information:**

The online version contains supplementary material available at 10.1186/s40560-020-00520-w.

## Background

As of October 2020, the severe acute respiratory syndrome coronavirus 2 (SARS-CoV-2) pandemic (COVID-19) has led to over 33 millions of infections [1]. About 25% of hospitalized patients required ICU admission for supportive care [2]. The rapid progression of COVID-19 and the dramatic escalation of cases have led to the lockdown of the population, as well as a strict limitation of access to hospitals and care facilities for relatives, in order to break the chain of transmission. Overnight, visits in our ICU have been prohibited by hospital policy. Before this unexpected crisis, our intensive care unit (ICU) was organized on a “24/7 open Unit” model with unrestricted visiting policy, as recommended by critical care societies [3].

Having a beloved or a family member hospitalized in ICU is a stressful event [4]. Relatives are suddenly immersed in “another world,” oscillate between fear and hope, and experience extreme vulnerability and helplessness. Twenty-five to 50% of the relatives would subsequently develop significant anxiety, depression, and stress symptoms [3] called “post-intensive care syndrome-family” (PICS-F) [4–8]. Taking time to provide clear information, listening to them, involving them in decision process and care, and giving families full access to the ICU may help reduce the prevalence of post-traumatic stress disorder [9, 10]. Particularly, unrestricted visits in ICU decrease anxiety and psychological disorders, allow for a better adhesion to medical decisions, and improve satisfaction on both impression of safety and quality of care for patients and relatives [7, 11].

Having to find a new way to communicate with and inform relatives, we committed to call families every day before 6 pm, and at any time in case of acute problem. We asked them to choose a reference person (RP) in order to facilitate communication. A reference person (RP) is a person designated among the patient’s next of kin during hospitalization in the ICU, in the absence of an officially designated surrogate [12, 13]. The call was conducted by either a resident or a senior doctor, and included daily news from the patient. As soon as possible after the patient woke up (even if still intubated), the phone was put on speaker or a video call was performed with a tablet so that the RP could speak to him/her. Yet, we felt that the total restriction of visits represented a significant additional psychological burden for relatives, likely to increase their potentially traumatic feeling of helplessness [14–16]—all the more in the context of high mediatization and generalized fear. At present, and although the ICU stay may be a traumatic experience for the RP, no psychological support is planned for RPs having a relative hospitalized in an ICU. Thus, unless the RP specifically asks for some support, she/he will not have a chance to get any. Our study suggests the relevance of psychological support—possibly also at a preventative level, before it is explicitly asked. It might thus be relevant to have a brief dual physician/psychologist exchange with RPs to explain that stress and anxiety are normal in such situations, and to propose a separate counseling consultation with a psychologist.

We thus decided to address the psychological suffering of relatives specific to this novel type of remote-only communication. To that effect, we focused on RPs, with whom all remote communications took place. While most work on the responsibility and distress associated with being a reference person focuses on decision making [17], our hypothesis was that RPs might experience higher levels of distress associated with having to be the sole intermediate between ICU caregivers and the rest of the family/close ones in context of COVID-19 pandemic, and that the remote-only communication might affect the RP’s communication with caregivers or family members. To our knowledge, this has not been explored in published literature.

We therefore evaluated RPs’ depression and anxiety symptoms, and their satisfaction, during ICU stay (admission and discharge) and at 3 months. During the 3-months assessment, we also examined additional elements: what it felt like to be the reference person in this remote context, the manner in which they transmitted the information, and the coping strategies they used to manage the situation. We then assessed the extent to which these additional elements could account for their anxiety and depression, during or after hospitalization.

## Methods

### Study participants

The RPs of all patients hospitalized in a French tertiary hospital ICU for a severe form of COVID-19 in May–June 2020 were included in a prospective study. Only one relative per patient—the RP designated by other family members—was included in the study and asked for consent to 3 phone interviews: at admission and discharge or patient death by an ICU physician, and 3 months after ICU admission by a clinical psychologist. Independently of their participation in this study, a daily phone call was given by ICU physicians to inform the RP of the patient’s clinical evolution. Non-inclusion criteria were refusal to participate in the study, and insufficient mastery of French language. The local ethical committee approved the study (NCT04385121).

### Interviews and measurements

Considering the context of COVID-19, all interviews were conducted by phone, by an ICU physician for daily calls during ICU stay and for the interviews at admission and discharge/death; at 3 months, interviews were conducted by a clinical psychologist experienced in working with ICU patients and families (incl. RPs) during the COVID pandemic. This psychologist was independent from the ICU team and unit in which the study took place.

During these interviews, the following scales were completed: (i) Hospital Anxiety and Depression Scale (HADS), which was performed within 72 h after admission (baseline), at ICU discharge or patient death, and 3 months after ICU admission; (ii) Impact of Events Scale-Revised (IES-R) at 3 months [18, 19]. The HADS was used to screen for anxiety and depressive disorders. It has 14 items, rated from 0 to 3. Seven questions are related to anxiety (total HADS-A) and seven others to depression (total HADS-D), thus providing 2 scores (maximum score for each subscale = 21 points) [18]. For the depression and anxiety subscales (HADS-D and HADS-A), scores of 8 or more were used for definite cases [20–22]. The revised version of the Impact of Event Scale (IES-R) has seven additional questions and a scoring range of 0 to 88. Scores exceeding 32 on IES-R scale were considered as indicating a post-traumatic stress disorder [20–22]. Responses to open-ended questions (see below), including alcohol/tobacco consumption, sleep quality, level of satisfaction, feeling of security, and confidence in caregivers, were also evaluated on a 5 or 10 points Likert-type scale at 3 months, so that the respondent could complete the answer with their own words or thoughts.

In addition, during the 3rd month interview, after the clinical psychologist helped RPs complete the questionnaires, she interviewed them in an open-ended semi-directive fashion about their experience of the situation [23]: that is, of being an RP in a context of remote-only, doctor-mediated communication. When necessary, she referred them to professional counseling. In this sequence, she asked two questions: “Q1. How did you experience being a reference person in this context, i.e., being an intermediate between the ICU and the rest of the family and/or close ones? Q2. How did you cope with being in that position?” She made sure these questions were understood as open-ended, and not as requiring categorical answers (e.g., “well vs. unwell”, etc.).

With Q1, we wanted to explore how the person experienced the specific responsibility associated with the context, and more generally how they handled communication with the rest of the family. With Q2, we sought to highlight the variety of coping strategies.

### Statistical analysis

As the study was observational and in the absence of possible pertinent comparative group (no alternative modality was possible/allowed for visits and communication), RPs for all patients admitted during the study period were included if they consented to participate. Descriptive analyses were performed indicating median and IQR for numeric variables, and frequencies for categorical variables. Inferential analysis investigating the relations between our variables were performed in accordance with the type of variables considered. Linear regressions were used when both the dependent and independent variables are continuous, and the dependent variable normally distributed. When the dependent variable has a natural left bound and can be considered as a count variable, Poisson regression was employed. In case the independent variable is categorical and binary, Student *T* test were performed. Finally, we used Chi-2 test when both the dependent and independent variables were categorical. All analyses were performed using R software version 4.0.2 (R Core Team 2020).

### Qualitative analysis

Interviews were transcribed verbatim, and data were analyzed by the second author, a clinical psychologist with clinical experience in ICU during the COVID pandemic (SC), and by the co-last author and co-supervisor of the study, an Associate Professor in Clinical Psychology experienced in qualitative research (OP).

A qualitative methodology was used for analysis. Qualitative methodologies are used to explore peoples’ experiences under various conditions in order to characterize a phenomenon when little prior work has been accomplished [23]: they are called for when questions are semi-directive, i.e., open-ended. This process entails the selection of seemingly relevant variables to be studied. Considering the length of the interviews (averaging about 35 min) and the type of questions, it is not surprising that the salient qualitative variables were not patterns across the data, but rather mostly remained within the answers to Q1 (overall experience) and Q2 (coping strategies) respectively.

We used a thematic approach, in line with previous studies [24, 25]. Each team member independently read all of the transcripts of the interviews with the RPs. They then met and offered what they considered to be meaningful, information-rich descriptions of RPs’ overall experience and coping strategies. Both descriptions were then sorted into themes, that is, patterns that are important to the description and understanding of a phenomenon. Differences of opinion about the themes and examples with respect to transcripts were discussed by raters during several analysis meetings. Findings from this process are reported as descriptive information, and a sample of quotes (with the status and sex of the RP) were selected to represent the themes. Final themes and examples of overall experience and coping strategies were agreed upon by all members of the analysis team.

### Quantitative and qualitative data integration

Emerging themes turned out to be qualitative (categorical) variables; this format allowed us to statistically relate them with quantitative HADS and IES-R scores by drawing on the statistical procedures described in the above section.

## Results

### RP characteristics

One hundred and one RPs were screened for inclusion during the study period. Thirteen RPs were excluded because of refusal to participate to the study (*n* = 5) and/or insufficient mastery of French language (*n* = 8). Eighty-eight RPs were included in the study. Most RPs were women (*n* = 57, 65%). Eighty-one RPs (92%) were first-degree relatives of the patient; Table 1 describes their demographic characteristics, and scores to the different numeric scales administered. All 88 RPs completed the interview at admission and ICU discharge/patient death. Among them, 33 RPs properly completed the long interview at 3 months; 9 other interviews were excluded for being quite poor (3 were extremely short, and 6 RPs had important language difficulties); 9 RPs did not honor their rendezvous; and 37 RPs declined, most frequently because of painful mourning or subsequent unwillingness to partake in the interview without further motive. The 6 painfully bereaved qualified as Persistent Complex Bereavement Disorder; a defense mechanism against the recently stressful situation was most likely at play in a vast proportion of both missed rendezvous and subsequent unwillingness, but lack of detailed data makes it hard to assess which. Thus, while it is impossible to assert exactly how many dropouts came from pre-existing or temporary mental condition, it seems reasonable to assert that at least a fraction of the RPs quit the study because of a pre-existing or temporary mental condition. In terms of background, only 5 RPs (about 15%) seemed to have a medical history of generalized anxiety disorder (GAD)—about 3 to 4 times more than general prevalence, depending on the methodology [26]. GAD was the main disorder: depressive traits were apparent in many RPs, but they did not appear connected with a pre-existing condition and instead seemed to be situation-dependent—depressive disorder had only been explicitly diagnosed in 2 RPs (about 6%). Thus, the gap between few prior psychological problems and relatively high anxiety-depression scores and overall negative experience (see just below) would seem to indicate that these strong psychological effects of remote-only communication are mostly situation-dependent.

**Table 1:** Characteristics of reference persons

		*N* = 88
Age	Median, SD	57 (15)
Gender: female	*n* (%)	57 (65)
Relationship to patient: first degree	*n* (%)	81 (92)
Spouse/partner		51 (58)
Grown child		22 (25)
Parent		5 (6)
Sister/brother		3 (3)
Other		7 (8)
Professional occupation	*n* (%)	
Working/studying		44 (50)
Unemployed		24 (27)
Retired		19 (22)
Sleep quality	10 points Likert-type scale	6 [4; 8]
Security feeling	10 points Likert-type scale	9 [7; 10]
Confidence feeling	10 points Likert-type scale	10 [9; 10]
Satisfaction feeling	10 points Likert-type scale	10 [9; 10]
Comprehension	10 points Likert-type scale	10 [8; 10]
Increased smoking^a^	5 points Likert-type scale	4 [3; 4]
Increased alcohol consumption^a^	5 points Likert-type scale	4 [3; 4]
Baseline HADS at ICU admission	Numeric score	23 [16; 31]
Anxiety/admission	Numeric score	13 [9; 16]
Depression / admission	Numeric score	10 [7; 13]
HADS at ICU discharge or death	Numeric score	16 [9; 21]
Anxiety/ICU discharge	Numeric score	8 [5; 12]
Depression/ICU discharge	Numeric score	7 [3; 11]

For count variables: sum and percentage. For numeric score variables and Likert-type scale variables: Median [IQR]

^a^1 point: completely agree; 2 points: somewhat agree; 3 points: somewhat disagree; 4 points: completely disagree; 5 points: no advice

Age, gender, relationship, and mortality did not differ whether the participant accepted to complete the interview or not. Supplementary table 1 describes the mortality rate for the patients and their ICU length of stay.

### Prevalence of anxiety and depression symptoms in RPs was high during ICU stay

**Fig. 1:**
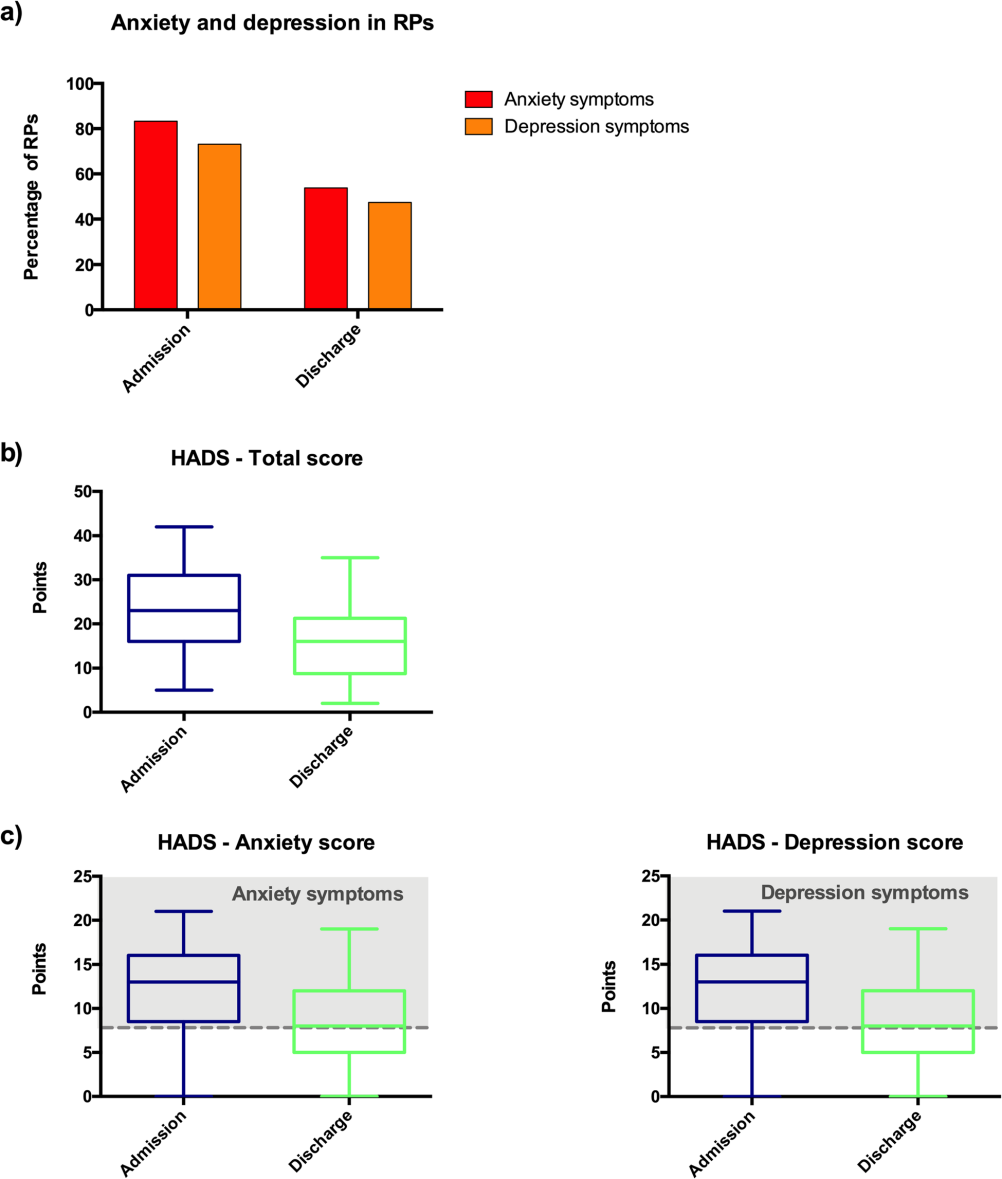
Anxiety and depression symptoms in reference persons at ICU admission and discharge. **a** Prevalence of anxiety and depression symptoms in reference persons (RPs) at ICU admission and discharge. **b** Evolution of total Hospital Anxiety and Depression Scale (HADS) between ICU admission and discharge in the 88 RPs, represented by whisker boxes (horizontal line inside the box: median, upper, and lower boxes: limits 25–75th percentiles and T-bars: 10–90th percentiles, respectively). **c** Evolution of the HAD anxiety and depression subscales between ICU admission and discharge in the 88 RPs. Scores of 8 points or more in each subscale were used for definite cases (gray dashed line) of anxiety and/or depression. Whisker boxes (horizontal line inside the box: median, upper and lower boxes: limits 25–75th percentiles and T-bars: 10–90th percentiles, respectively)

In this harsh context of COVID-19 pandemic, with visit prohibition and remote-only communication, reference persons suffered from considerable psychological distress: prevalence of anxiety and depression symptoms among RPs was indeed of 83% and 73% respectively in the 72 h following patient admission (Fig. 1a), and these symptoms were mostly (*n* = 87, 99%) attributed to COVID-19 diagnosis announcement and ICU admission. These anxiety and depression symptoms decreased to 54% and 47% respectively at patient discharge/death (Fig. 1a).

**Fig. 2:**
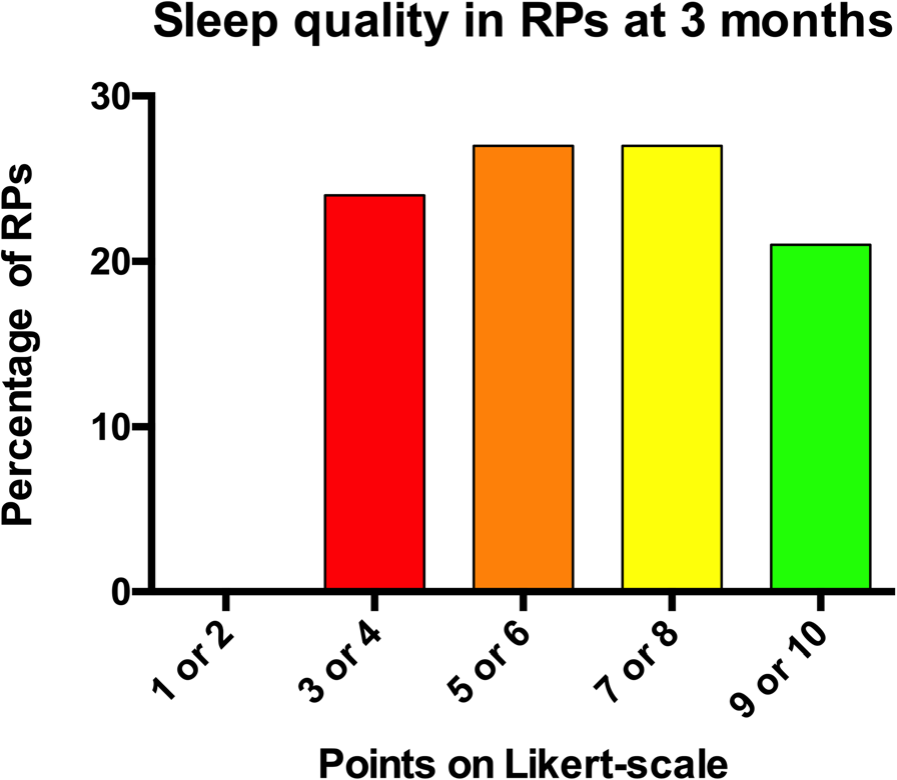
Sleep quality in reference persons at 3 months. Sleep quality was evaluated on a 10 points Likert-type scale at 3 months: 1 being a very bad quality of sleep and 10 a very good one

Median HADS significantly decreased between ICU admission and discharge/death, with a decrease in both anxiety and depression symptoms (*p* < 0.01 for all comparisons) (Fig. 1b, c). During the first 3 months, lower HADS decrease was associated with patient death or the patient being still hospitalized at 3 months and sleeping disorders in RPs (*p* < 0.01). HADS evolution was influenced neither by the type of relationship (romantic [spouse or significant other] vs. familial), nor by being also diagnosed with COVID-19 or having other relatives with COVID-19. Evolution of anxiety symptoms was neither influenced by the feeling of security, nor by confidence in caregivers and level of satisfaction. Depression symptoms were higher if the patient has died and/or the RP suffered from sleeping disorders (*p* < 0.01), and were associated with increased tobacco consumption (*p* < 0.05). Sleep quality at months in RPs are reported on Fig. 2.

### RPs reported high levels of security feeling, confidence, and satisfaction

**Fig. 3:**
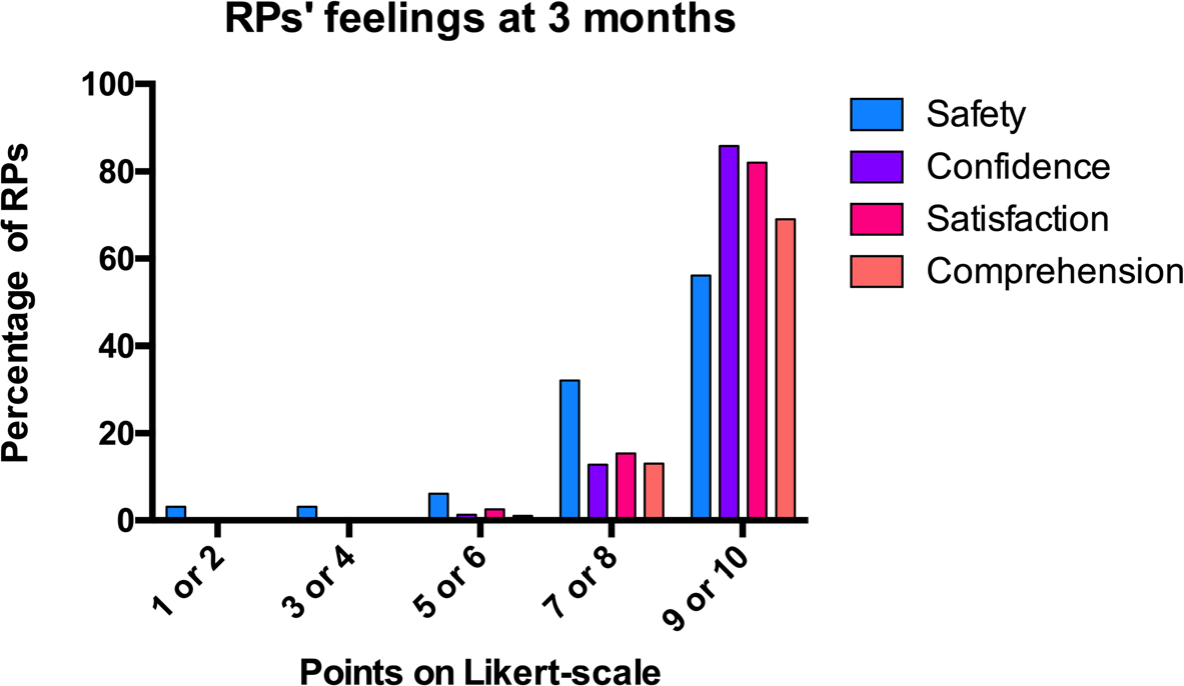
Evaluation of reference persons’ feelings at 3 months. Feelings of (i) safety, (ii) confidence in intensive care unit (ICU) caregivers, therapeutic decisions and information, (iii) satisfaction toward ICU care and information modalities, and (iv) comprehension of diagnosis, therapeutic, and prognosis during ICU stay. These were evaluated on a 10 points Likert-type scale at 3 months: 1 being a very bad feeling of security/confidence/satisfaction/comprehension and 10 a very good one

Despite visit prohibition, the vast majority of RPs (*n* = 87, 99%) felt that the patient was safe (9 [7; 10] points/1–10 Likert scale). They also felt confident with caregivers, therapeutic decisions, and information provided (10 [9; 10] points /1-10 Likert scale) and were satisfied with the manner and frequency with which information was provided (10 [9; 10] points/1–10 Likert scale) (Fig. 3). RPs having already experienced prior hospitalizations of the patient in non-COVID-19 context—whether in ICU or not—stated that they had never benefited from daily news directly from the medical team; they were grateful of this.

### RPs reported an overall negative experience of being an RP in a remote-only context

Qualitative analysis of these interviews showed that themes were not patterns across the data, but mostly remained within the answer to each question. We thus present thematic results below each question. Quotation marks refer to verbatim extracts.

As could be expected with Q1 (*How did you experience being a reference person in this context*, i.e., *being an intermediate between the ICU and the rest of the family and/or close ones*?), all RPs stressed the specific type of “responsibility” associated with being an RP in a remote-only context. Interestingly, the 3 most salient themes turned out to be dichotomous variables, which tell us whether each theme was addressed or not by each participant.

Theme 1: Of all 33 RPs, 24 (73%) reported an *overall negative subjective experience* of being the intermediate between the ICU and the rest of the relatives in this remote-only context, in which they were the only ones who heard, received and were able to disclose information to other relatives. Nine RPs (27%) had an overall *positive experience* of being an RP in this context.

Most of those who had a positive experience said it made them feel “in control” (daughter) and “important,” and because they were sure to receive daily, “firsthand information” (son) from ICU teams which they “fully trusted” (female spouse) because they did “a really good job” (female spouse).

Most of those who had a negative experience mentioned the responsibility with respect to other relatives (having to remember the information, and to disclose bad news), even more so when “they had no choice but to accept” (sister) being an RP. For some, the “anxiety-inducing” perspective of the daily call (female spouse; daughter; male spouse; father) turned the period into a “daily nightmare” (female spouse) or a “2-month nightmare” (female spouse), sometimes including hallucinatory episodes (feelings of “telepathic communication” (female spouse)). The “self-denial” of the seriousness of the disease made being a RP very distressing; sometimes, RPs felt “guilty” of having transmitted the disease even though it was medically unlikely (father). When the relative was admitted to the ICU, “not being able to say goodbye or even to remove the relative’s wedding ring” was extremely painful (male spouse). Interestingly, some underlined that they “experienced the disease as something fatal, against which doctors could not do much” (son): while this added to their overall helplessness, they still felt that doctors did all they could.

Theme 2: Most RPs (*n* = 22, 67%) adopted a *narrow information diffusion strategy*, restricting the array of contacted relatives to a very few and/or only contacting them rarely, while 33% of them adopted a *wide information diffusion strategy* (11 RPs, 33%).

Narrow diffusion strategies mainly emerged because “repeating everything everyday was becoming increasingly hard” (son)—a statement made by many. Often, RPs relied on technological devices themselves in order to avoid direct interaction: “I created a PDF document and shared it on Google because it saved me from doing the calls” (female spouse); “I wrote a little SMS and copy-pasted it to all relatives. I could not call everyone: it was too emotionally hard” (female spouse); a female spouse said “I forced myself to text the in-laws every 10 days … I could not do more.”

Theme 3: Only 10 RPs (30%) spontaneously related the situation to a *prior traumatic experience*, while 23 (70%) did not. A son said “I find it hard to accept all of this ... it summoned the death of my mother (10 years ago).” A female spouse said her husband “had the same symptoms—lung clots—as [her] father, who died of cardiac arrest from pneumonia.” Interestingly, the type of trauma was not necessarily a disease or the loss of a close one: referring to hospitalization and the fear of losing his spouse, a husband stressed “it was the same fear I felt when meeting a burglar at home.” Some RPs also mentioned previous traumatic experiences (undisclosed fatal disease of a relative, death of twins at birth), but primarily in relation to their current “need to know” (female spouse), i.e., to receive information. Another female spouse “preferred to know for herself to be able to protect the children.”

### Variety of coping strategies among RPs

With Q2 (*How did you cope with being in that position*?), we sought to collect the variety of coping strategies; all respondents understood the question in this way. These activities and strategies could not easily be separated along axes such as solitary vs. gregarious activity, leisure vs. work, physical vs. mental activity: many mentioned both “work” and “leisure” (sister), both “remaining alone” and also “getting in touch with relatives” (daughter; female spouse) however punctually, both “reading” and “going for long, tiring walks.” As could be expected, faith and religion appeared in numerous interviews, sometimes as “reassuring” (female spouse) and sometimes as “ways to be in touch” (male spouse) with the relative. Several mentioned activities oriented toward the future return of the patient, such as “cleaning” and “preparing the house” (male, spouse; male, spouse): “I did some painting and the mechanics he wanted to do. He will come home and he will see that I did, it will be a surprise” (female, spouse); “I started cooking for her.”

It is interesting to note that in a subset of the 33 RPs, a gap can be observed between their scores at the standardized scales and their spontaneous recollection of the painful stay of their relative in the ICU. This is partial evidence of the relevance of semi-structured, open-ended qualitative methods in comparison with the completion of quantitative scales. We could hypothesize that open-ended questions tend to bring about more spontaneous answers, which could be closer to people’s actual behaviors and attitudes, while standardized scales might induce a more distanced perspective, further from “the heat of the moment.”

There was also a significant link between having an overall negative subjective experience and preferring narrow diffusion strategies (*p* < 0.01). All RPs who negatively experienced the situation also employed a restraint diffusion strategy (100%). Conversely, the majority of RPs who had an overall positive experience of being an RP used a wide diffusion strategy (82%). On the basis of the interviews, we could assume that the wide diffusion strategy partially explains overall positive experience: wide diffusion strategies were often described as a way to feel less powerless.

At the end of the interview, 8/33 relatives (24%) were referred to professional counseling support.

### PTSD prevalence at 3 months was prominent among RPs

Supplementary table 2 describes the patients’ mortality rate for the 33 RPs who completed the interview, the presence of COVID-19 symptoms in the RP or in his/her family members, the numeric scores obtained at the IES-R, and the three qualitative variables obtained from the interviews.

**Fig. 4:**
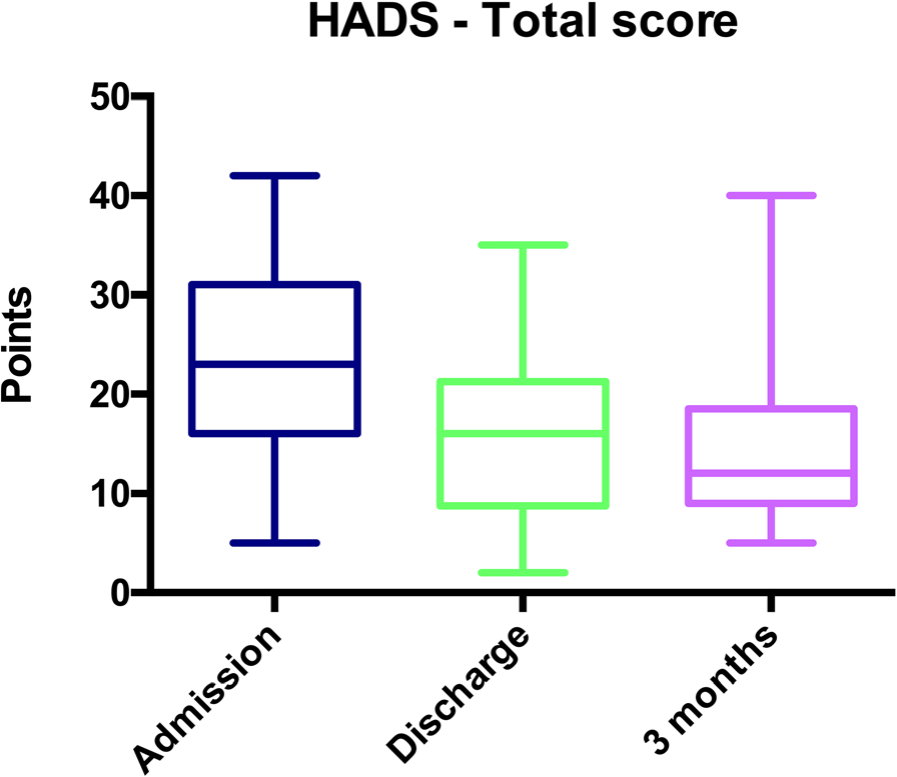
Anxiety and depression symptoms in reference persons at ICU admission, discharge and at 3 months. Hospital Anxiety and Depression Scale (HADS) evolution between admission, discharge/death, and 3 months later, in 33 RPs, represented by whisker boxes (horizontal line inside the box: median, upper and lower boxes: limits 25–75th percentiles and T-bars: 10–90th percentiles, respectively)

For the subgroup of 33 respondents, HADS evolution on the three measurement occasions (admission, discharge, or death, interview 3 months later) is depicted in Fig. 4. For the subgroup which completed the 3rd-month interview, median HADS was 12 [9; 18] points. A repeated-measures ANOVA revealed that HADS significantly decreased with time (*p* < 0.01). Post-hoc analysis with Bonferroni correction revealed a difference between ICU admission and ICU discharge or death (*p* < 0.01), and no difference between ICU discharge or death and 3 months later (*p* = 0.71).

The relations between quantitative variables (HADS and IES-R score) and qualitative variables (subjective experience, diffusion strategy, and prior traumatic experience) were also obtained from interview analysis. Median HADS was higher in RPs who had a negative experience of being an RP in a remote-only context (*p* < 0.01; median 16 vs. 11 points). HADS was not influenced by the chosen diffusion strategy (*p* = 0.67), nor by the explicit mention of a prior traumatic experience (*p* = 0.80).

**Fig. 5:**
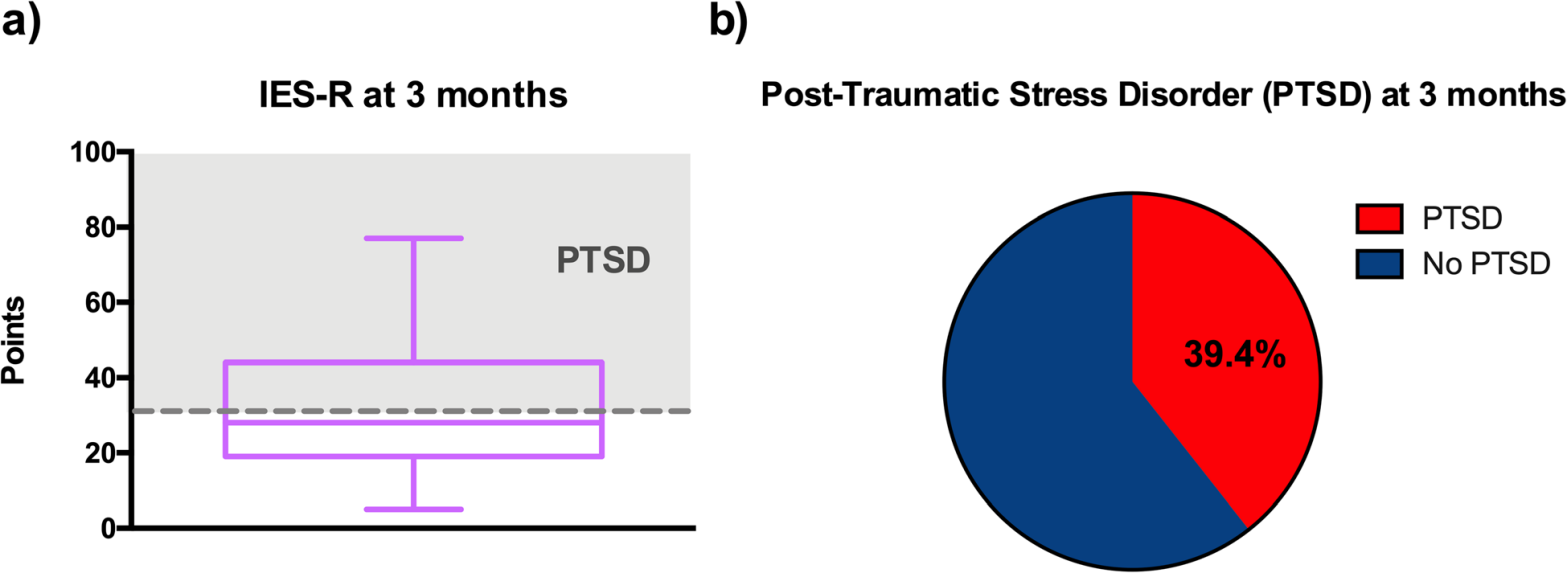
Post-traumatic stress disorder in reference persons at 3 months. **a** Impact of Event Scale-revised (IES-R) represented by whisker boxes (horizontal line inside the box: median, upper, and lower boxes: limits 25–75th percentiles and T-bars: 10–90th percentiles, respectively). Scores exceeding 32 points (dashed line) on IES-R scale were considered as indicating a PTSD. **b** Prevalence of post-traumatic Stress Disorder (PTSD) in reference persons (RPs) at 3 months

Prevalence of post-traumatic stress symptoms (risk of PTSD) at 3 months was 39% in the subgroup of 33 RPs, with a median IES-R of 28 [19; 42] points (Fig. 5a, b). Median IES-R was higher in RPs who had a negative experience of the ICU stay (*p* < 0.01; median 35 vs. 23) and who did not develop a wide diffusion strategy (*p* < 0.01; median 34 vs. 29). IES-R scores were not influenced by the explicit correlation of the situation with a prior traumatic experience (*p* = 0.92).

## Discussion

In this article, we describe the psychological effects of remote-only communication among RPs of ICU patients during the COVID-19 pandemic. The major results of this study are (i) a high prevalence of anxiety and depression symptoms during ICU stay, (ii) a high prevalence of PTSD at 3 months, and (iii) an overall negative experience of being an RP in remote-only context, with having to support the weight of a “specific” responsibility. The rapid progression of COVID-19 pandemic and the massive influx of patients in our hospital have led to ICU visit prohibition overnight, and this situation has been encountered in many ICUs facing such massive afflux of patients in France and Europe. To face this exceptional situation, a chosen relative (the RP) was called daily by the medical team to provide information, preserve the link between relatives and caregivers, and try to make both RPs and other relatives feel less overwhelmed and helpless. As medical support might prevent post-traumatic stress disorder and facilitate potential future bereavement [27, 28], this major change in the usual communication channels with families warranted a specific examination—even more so since it cannot be ruled out that diseases leading to this type of remote-only communication will happen again in the future.

Consistent with studies conducted in non-COVID-19 ICU context [9, 10], RPs interviewed in our study experienced major psychological distress (median HADS-A > 8 points) and secondary high prevalence of PTSD at 3 months; this can plausibly be traced back to the successive announcement of COVID-19 diagnosis and worsening requiring ICU admission, to the forced remoteness, but maybe also to the responsibility of being an RP in this remote-only context, as highlighted by all RPs during their interviews. It is likely that restricted visit policies, COVID-19 pandemic itself, and the impact of lockdown and related frustrations have led to additional burden for RPs [14–16] and therefore represent independent confounding factors for the prevalence of depression and anxiety symptoms among RPs of ICU patients. These confounding factors cannot be fully individually isolated, in spite of the partial correlation we have evidenced between quantitative and qualitative variables (namely IES-R scores and negative experiences of being an RP, along with narrow diffusion strategies).

Interestingly, even though symptoms of anxiety and depression increased with the death of the patient, the feeling of security and the RPs’ levels of satisfaction and confidence in the healthcare team remained high. Even in cases of fatal evolution, RPs and relatives were thankful and satisfied with the daily contact and the global care. Yet, a few RPs indicated that their own bereavement process was complicated because of their intermediary status, requiring them to constantly pass on information in a context of high uncertainty, the constant evolution of which made it harder to prepare themselves for a potential loss. It is however difficult to draw more conclusions regarding bereaved family members’ experience from our qualitative data since the process of bereavement is best studied after 6 months [22], and would benefit from a larger sample. Previous studies, prior to COVID-19 pandemics, consistently reported better surrogates’ ratings of the quality of communication when a multicomponent family-support intervention was implemented compared to standard of care [17]. Although the third month interview was performed by a clinical psychologist independent from the ICU team and unit, we cannot rule out that the lack of anonymity may have biased the responses on satisfaction/confidence in the ICU team involved in the care of a relative; this could, in part, account for the observed gap between the scores and the open questions.

The remote-only communication model that we used in a French COVID-19 epicenter during the hard times of the pandemic therefore seems to effectively preserve a minimal and reassuring link between caregivers and RPs of ICU patients. Calling RPs once a day represented a considerable workload (mental and otherwise) for medical teams, all the more since such calls were often extremely emotionally laden. Calls often turned out to be prolonged, requiring active listening, for which ICU physicians were not specifically trained. This is the reason why the 3-month interview was performed by a trained clinical psychologist with experience in COVID-19 ICU contexts, but independent from the ICU team.

One can assume that distress symptoms might have been more prominent without any alternative means of communication (here, the remote-only communication) with families. For example, some RPs called us back to report their major concern at ICU discharge (in ward or transfer to another ICU), because they suddenly ceased to receive information as all caregivers were overwhelmed by massive patient afflux and could not devote much time to family information.

Although being an RP could be considered as a coping strategy to face this exceptional situation, most RPs reported it as an overall negative experience. Indeed, being the sole interlocutor of the medical team meant that RPs were also the only ones who could understand and disclose information to the other relatives, and this added supplementary burden to the distress of critical illness. Most RPs thus described narrow diffusion strategies of information as a consequence of this burden. While separation can induce post-traumatic stress disorders in relatives [14], not much has been published regarding the post-traumatic effects of daily remote contacts. Yet, the rates of post-traumatic stress symptoms (IES-R) match those found by studies of relatives in non-remote contexts [28], which could be due to a high degree of interaction with the medical team in an attempt to compensate for remoteness.

To the best of our knowledge, it is the first time that a fully remote communication model consisting in virtual meetings, without direct telehealth implications, has been used in an ICU for such a long period and with such a high number of severe patients. Our study is descriptive and has no comparator group. We can therefore not formally relate the anxiety and depression symptoms to remote communication. Furthermore, the third month follow-up is certainly underpowered due to the high rate of non-interpretable interviews or refusals at this stage. Consistent with previous studies [24, 25], the use of mixed methods to feed clinical practice shows the added benefits of qualitative approaches: they allowed for an inductive exploration (required in novel clinical contexts) which in turn generated categories significantly correlated with measures of distress (IES-R). Thus, paying attention to elements displayed in informal exchanges (regarding the type of diffusion strategy, or the overall experience) can help caregivers accurately grasp the different dimensions of such distress.

The major psychological distress described in our study was somewhat expected, but highlights that considerable efforts should be made to improve communication with families, especially in such an anxiety-inducing pandemic context. It also highlights the necessity to develop alternative means to disclose information to families and maintain contact between patients and families in exceptional situations, drawing on new technologies. Our approach may contribute to help caregivers improve their support to relatives [17] and, in particular, RPs. In such remote-only contexts, the determinants and modalities of such a support largely remain to be devised: this present paper was also meant as an initial contribution to this emerging field of research. Further studies about the communicational and relational skills required by this type of remote daily contact should motivate institutional or even national recommendations in times of exceptional crisis, in order to encourage clinicians to also care for the relatives, and in particular RPs, who stand in-between caregivers and the rest of the family.

## Conclusions

Reference persons of ICU patients admitted for COVID-19 experienced considerable psychological distress, with high prevalence of anxiety and depression during ICU stay, and prominent post traumatic distress syndrome at 3 months. Being an RP in a context of remote-only communication was considered as a specific responsibility and qualified as an overall negative experience.

## Supplementary Information

**Additional file 1.**

**Additional file 2.**

## Data Availability

All data generated or analyzed during this study are included in this published article and its supplementary information files.

## Abbreviations

HADS: Hospital Anxiety and Depression Scale
ICU: Intensive care unit
IES-R: Impact of Events Scale-Revised
PICS-F: Post-intensive care syndrome-family
RP: Reference person
SARS-CoV-2: Severe acute respiratory syndrome coronavirus 2

## References

[1] COVID-19 Dashboard by the Center for Systems Science and Engineering (CSSE) at Johns Hopkins University (JHU) - Coronvirus Ressource Center. https://coronavirus.jhu.edu/map.html.

[2] Phua J, Weng L, Ling L, Egi M, Lim CM, Divatia JV (2020). Intensive care management of coronavirus disease 2019 (COVID-19): challenges and recommendations. Lancet Respir Med..

[3] Davidson JE, Aslakson RA, Long AC, Puntillo KA, Kross EK, Hart J (2017). Guidelines for Family-Centered Care in the Neonatal, Pediatric, and Adult ICU. Crit Care Med..

[4] Lautrette A, Darmon M, Megarbane B, Joly LM, Chevret S, Adrie C (2007). A communication strategy and brochure for relatives of patients dying in the ICU. New Engl J Med..

[5] Davidson JE, Jones C, Bienvenu OJ (2012). Family response to critical illness: postintensive care syndrome-family. Crit Care Med..

[6] Adelman RD, Tmanova LL, Delgado D, Dion S, Lachs MS (2014). Caregiver burden: a clinical review. Jama..

[7] Garrouste-Orgeas M, Philippart F, Timsit JF, Diaw F, Willems V, Tabah A (2008). Perceptions of a 24-hour visiting policy in the intensive care unit. Crit Care Med..

[8] TSP I (1995). A controlled trial to improve care for seriously ill hospitalized patients. The study to understand prognoses and preferences for outcomes and risks of treatments (SUPPORT). The SUPPORT Principal Investigators. Jama..

[9] Kose I, Zincircioglu C, Ozturk YK, Cakmak M, Guldogan EA, Demir HF (2016). Factors affecting anxiety and depression symptoms in relatives of intensive care unit patients. J Intensive Care Med..

[10] Garrouste-Orgeas M, Vinatier I, Tabah A, Misset B, Timsit JF (2016). Reappraisal of visiting policies and procedures of patient's family information in 188 French ICUs: a report of the Outcomerea Research Group. Ann Intensive Care..

[11] Rosa RG, Tonietto TF, da Silva DB, Gutierres FA, Ascoli AM, Madeira LC (2017). Effectiveness and safety of an extended ICU visitation model for delirium prevention: a before and after study. Crit Care Med..

[12] Quenot JP, Meunier-Beillard N, Ksiazek E, Abdulmalak C, Berrichi S, Devilliers H (2020). Criteria deemed important by the relatives for designating a reference person for patients hospitalized in ICU. J Crit Care..

[13] Rigaud JP, Hardy JB, Meunier-Beillard N, Devilliers H, Ecarnot F, Quesnel C (2016). The concept of a surrogate is ill adapted to intensive care: criteria for recognizing a reference person. J Crit Care..

[14] Akgun KM, Shamas TL, Feder SL, Schulman-Green D (2020). Communication strategies to mitigate fear and suffering among COVID-19 patients isolated in the ICU and their families. Heart Lung..

[15] Montauk TR, Kuhl EA (2020). COVID-related family separation and trauma in the intensive care unit. Psychol Trauma..

[16] Lissoni B, Del Negro S, Brioschi P, Casella G, Fontana I, Bruni C (2020). Promoting resilience in the acute phase of the COVID-19 pandemic: Psychological interventions for intensive care unit (ICU) clinicians and family members. Psychol Trauma..

[17] White DB, Angus DC, Shields AM, Buddadhumaruk P, Pidro C, Paner C (2018). A Randomized trial of a family-support intervention in intensive care units. New Engl J Med..

[18] Zigmond AS, Snaith RP (1983). The hospital anxiety and depression scale. Acta Psychiatr Scand..

[19] Weiss D, Marmar C, Wilson JP, Keane TM (1997). The impact of event scale–revised. Assessing psychological trauma and PTSD.

[20] Garrouste-Orgeas M, Flahault C, Vinatier I, Rigaud JP, Thieulot-Rolin N, Mercier E (2019). Effect of an ICU diary on posttraumatic stress disorder symptoms among patients receiving mechanical ventilation: a randomized clinical trial. Jama..

[21] Kalfon P, Alessandrini M, Boucekine M, Renoult S, Geantot MA, Deparis-Dusautois S (2019). Tailored multicomponent program for discomfort reduction in critically ill patients may decrease post-traumatic stress disorder in general ICU survivors at 1 year. Intensive Care Med..

[22] Kentish-Barnes N, Chevret S, Champigneulle B, Thirion M, Souppart V, Gilbert M (2017). Effect of a condolence letter on grief symptoms among relatives of patients who died in the ICU: a randomized clinical trial. Intensive Care Med..

[23] McAdam JL, Arai S, Puntillo KA (2008). Unrecognized contributions of families in the intensive care unit. Intensive Care Med..

[24] Kentish-Barnes N, McAdam JL, Kouki S, Cohen-Solal Z, Chaize M, Galon M (2015). Research participation for bereaved family members: experience and insights from a qualitative study. Crit Care Med..

[25] Murphy MR, Escamilla MI, Blackwell PH, Lucke KT, Miner-Williams D, Shaw V (2007). Assessment of caregivers' willingness to participate in an intervention research study. Res Nurs Health..

[26] Pratt LA, Brody DJ. Depression in the U.S. household population, 2009-2012. NCHS Data Brief. 2014;(172):1–8. 25470183

[27] Morris SE, Moment A, Thomas JD (2020). Caring for bereaved family members during the COVID-19 pandemic: before and after the death of a patient. J Pain Symptom Manage..

[28] Azoulay E, Pochard F, Kentish-Barnes N, Chevret S, Aboab J, Adrie C (2005). Risk of post-traumatic stress symptoms in family members of intensive care unit patients. Am J Respir Crit Care Med..

