# Supplementary material for: Psychological effects of remote-only communication among reference persons of ICU patients during COVID-19 pandemic
Source: J Intensive Care. 2021 Jan 9;9:5. doi: 10.1186/s40560-020-00520-w (PMC7794617; doi:10.1186/s40560-020-00520-w)
Supplement: Supplementary file 1 — Additional file 1. [file 40560_2020_520_MOESM1_ESM.docx]

**Supplementary table 1:**

|  | **N=88** |
| --- | --- |
| **Patient characteristics** |  |
| SAPS II (points) - median [IQR] | 49 [38; 64] |
| SOFA admission (points) - median [IQR] | 7 [4; 9] |
| Mortality-rate – n (%) | 18 (21%) |
| ICU LOS - median [IQR] | 11 [8; 19] |

ICU: intensive care unit; IQR: interquartile range; LOS: length of stay; SAPS II: simplified acute physiology score II; SOFA: Sepsis-related Organ Failure Assessment.
