# Supplementary material for: Psychological effects of remote-only communication among reference persons of ICU patients during COVID-19 pandemic
Source: J Intensive Care. 2021 Jan 9;9:5. doi: 10.1186/s40560-020-00520-w (PMC7794617; doi:10.1186/s40560-020-00520-w)
Supplement: Supplementary file 2 — Additional file 2. [file 40560_2020_520_MOESM2_ESM.docx]

**Supplementary table 2:** Characteristics of the 33 relatives who completed the interview at 3 months

|  | **Measurement** | **n=33** |
| --- | --- | --- |
| **Mortality rate** | N (%) | 7 (21%) |
| **COVID-19 symptoms in the relative** | N (%) | 12 (37%) |
| **COVID-19 in (other) relative’s family members** | N (%) | 12 (37%) |
| **IES-R** | Numeric score | 28 [19; 42] |
| **Subjective experience (positive vs. negative)** | Count of individual thematic occurrence during interview (positive) | 9 (27%) |
| **Diffusion strategies (large vs. narrow)** | Count of individual thematic occurrence during interview (large) | 11 (33%) |
| **Traumatic experience (presence vs. absence)** | Count of individual thematic occurrence during interview | 10 (30%) |

IES-R: Impact of Event Scale – revised.
